# Third‐line or above anlotinib in relapsed and refractory small cell lung cancer patients with brain metastases: A post hoc analysis of ALTER1202, a randomized, double‐blind phase 2 study

**DOI:** 10.1002/cai2.43

**Published:** 2023-01-17

**Authors:** Ying Cheng, Qiming Wang, Kai Li, Jianhua Shi, Baohui Han, Lin Wu, Gongyan Chen, Jianxing He, Jie Wang, Haifeng Qin, Xiaoling Li

**Affiliations:** ^1^ Department of Thoracic Medical Oncology Jilin Cancer Hospital Changchun China; ^2^ Department of Internal Medicine Affiliated Cancer Hospital of Zhengzhou University, Henan Cancer Hospital Zhengzhou China; ^3^ Department of Pulmonary Oncology Tianjin Medical University Cancer Hospital Tianjin China; ^4^ Department of Medical Oncology Linyi Cancer Hospital Linyi China; ^5^ Department of Respiratory Medicine, Shanghai Chest Hospital Shanghai Jiaotong University Shanghai China; ^6^ Department of Thoracic Medical Oncology Hunan Cancer Hospital Changsha China; ^7^ Department of Respiratory Medicine Harbin Medical University Cancer Hospital Harbin China; ^8^ Department of Thoracic Surgery The First Affiliated Hospital of Guangzhou Medical University Guangzhou China; ^9^ Department of Thoracic Medical Oncology Cancer Hospital Chinese Academy of Medical Sciences Beijing China; ^10^ Department of Pulmonary Oncology The Fifth Medical Centre of Chinese PLA General Hospital Beijing China; ^11^ Department of Medical Oncology Liaoning Cancer Hospital and Institute Shenyang China

**Keywords:** anlotinib, angiogenesis inhibitors, brain metastasis, advanced small cell lung cancer, safety

## Abstract

**Background:**

The prognosis of patients with small cell lung cancer (SCLC) and brain metastases (BM) was poor. This study aimed to explore the efficacy and safety of anlotinib as third‐line or above treatment in SCLC with BM.

**Methods:**

This was a subgroup analysis of the ALTER1202 trial, which was a randomized, placebo‐controlled trial aimed to evaluate the role of anlotinib as third‐line treatment or above in patients with SCLC. This study included patients with BM at baseline. The efficacy and safety outcomes included progression‐free survival (PFS), overall survival (OS), central nervous system (CNS), objective response rate (ORR), CNS disease control rate (DCR), time to CNS progression, and adverse events (AEs).

**Results:**

Twenty‐one and nine patients with BM were included in the anlotinib and placebo groups, respectively. The median PFS and OS were 3.8 months (95% confidence interval [CI]: 1.8–6.1) and 6.1 months (95% CI: 4.1–8.0) in the anlotinib group. Anlotinib was associated with a significant improvement in PFS (hazard ratio [HR] = 0.15, 95% CI: 0.04–0.51, *p* = 0.0005) and OS (HR = 0.26, 95% CI: 0.09–0.73, *p* = 0.0061) than placebo. Anlotinib significantly prolonged the time to CNS progression (*p* < 0.0001). The anlotinib group had a higher CNS DCR than placebo (95.2% vs. 22.2%, *p* = 0.0001). The most common grade 3 or higher AEs were increased lipase (19.0%), hypertension (14.3%), and hyponatremia (14.3%) in the anlotinib group.

**Conclusions:**

Anlotinib proved to have potential CNS activity and a manageable toxicity profile in patients with SCLC and BM, significantly delaying CNS progression.

## Graphical abstract

In this post hoc analysis of a multicenter, randomized, double‐blind, placebo‐controlled trial, anlotinib demonstrated a potential survival benefit in small cell lung cancer (SCLC) with brain metastases (BM) compared with placebo, with manageable toxicity. Anlotinib significantly prolonged median progression‐free survival (PFS) (hazard ratio [HR] = 0.15, 95% CI: 0.04–0.51; *p* = 0.0005) and median OS (HR = 0.26, 95% CI: 0.09–0.73; *p* = 0.0061) in SCLC patients with BM.

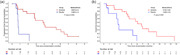

**Abbreviations**
AEs: adverse events
BM: brain metastases
CI: confidence interval
CIR: cumulative incidence rates
CNS: central nervous system
CR: complete response
CT: computed tomography
DCR: disease control rate
ECOG: Eastern Cooperative Oncology Group
FGFR: fibroblast growth factor receptor
HR: hazard ratio
MRI: magnetic resonance imaging
NCI‐CTCAE: National Cancer Institute Common Terminology Criteria for Adverse Events
NCR/NPD: non‐CR/non‐PD
NE: not evaluable
NSCLC: non‐small cell lung cancer
NTLs: non‐targeted lesions
ORR: objective response rate
OS: overall survival
PD: progressive disease
PDGFR: platelet‐derived growth factor receptors
PFS: progression‐free survival
PR: partial response
RECIST 1.1: Response Evaluation Criteria In Solid Tumors version 1.1
SCLC: small cell lung cancer
SD: stable disease
SRT: stereotactic radiotherapy
TKI: tyrosine kinase inhibitor
TLs: target lesions
TTBP: time to brain progression
VEGFR: vascular endothelial growth factor receptor
WBRT: whole‐brain radiotherapy

## 1 BACKGROUND

Small cell lung cancer (SCLC) is regarded as one of the most malignant pulmonary neuroendocrine neoplasms due to its increased recurrence and metastatic rates, which accounts for approximately 15% of lung cancer and causes about 250,000 deaths globally each year [1, 2, 3]. Approximately 10% of SCLC patients with their first diagnosis have already developed brain metastases (BM), and 50%–80% develop BM within 2 years of diagnosis [4, 5], seriously affecting the survival and quality of life of the patients [6]. In SCLC patients with BM, standard therapies such as whole‐brain radiotherapy (WBRT), systemic chemotherapy, and stereotactic radiotherapy (SRT) are commonly used [7]. Nevertheless, there is still a low overall survival (OS) rate, and the main chemotherapeutic tools are unchanged [8, 9, 10, 11].

As a result of immunotherapy, treatment has revolutionized a wide range of malignancies in recent years. Survival outcomes of immunotherapy in metastatic SCLC have been investigated through several large trials. The phase 1/2, open‐label CheckMate‐032 trial revealed a promising outcome of a combination of nivolumab and ipilimumab in patients with extensive‐stage SCLC, but BM patients were not included in the study [12]. Besides, in KEYNOTE‐028 and KEYNOTE‐158 studies, pembrolizumab as a third‐line or later treatment demonstrated encouraging antitumor effects with a lasting clinical benefit in SCLC patients. Despite the inclusion of patients with BM, a specific subgroup analysis was not conducted [13]. The CASPIAN study showed significantly better OS and progression‐free survival (PFS) of durvalumab plus chemotherapy than chemotherapy alone in the first‐line treatment of extensive‐stage SCLC [14]. In the IMpower‐133 study, the addition of atezolizumab to chemotherapy brings additional benefits for extensive‐stage SCLC in the first‐line setting [15]. Meanwhile, according to the subgroup analysis of these studies, therapeutic efficacy in the BM population needs to be further improved [14, 15].

Anlotinib is an oral tyrosine kinase inhibitor (TKI) targeting vascular endothelial growth factor receptor (VEGFR), platelet‐derived growth factor receptors (PDGFR), fibroblast growth factor receptor (FGFR), and c‐kit [16]. In a previous study, anlotinib was shown to provide benefit to patients with advanced non‐small cell lung cancer (NSCLC) accompanied by BM, with high efficacy in intracranial lesions [17]. Furthermore, the phase II multicenter, double‐blind, placebo‐controlled, randomized trial (ALTER1202) of anlotinib as a third‐line or later therapy in SCLC was also conducted [18, 19]. Due to its significantly improved median PFS and OS as well as the tolerable toxicity profile, anlotinib has been approved by the National Medical Products Administration for the third‐line treatment of SCLC in China.

This post hoc analysis included patients with BM at baseline in the ALTER1202 study, and aimed to evaluate the effectiveness and safety of anlotinib in patients with SCLC accompanied by BM to provide insights into clinical treatment.

## 2 MATERIALS AND METHODS

### 2.1 Study design and patients

This was a subgroup analysis of ALTER1202 (ClinicalTrial.gov #NCT03059797), which was a multicenter, randomized, double‐blind, placebo‐controlled phase 2 study aimed to evaluate the efficacy and safety of anlotinib as a third‐line treatment or above in patients with SCLC. The ALTER1202 study followed the Declaration of Helsinki and Good Clinical Practice Guidelines and was approved by the institutional review board of each participating center (approval number: 201701‐002‐01). All patients provided signed informed consent before any procedure. Eligible patients were randomly assigned at a 2:1 ratio to receive oral anlotinib 12 mg or placebo daily for 14 days every 3 weeks. A detailed study design has been published before [19].

The inclusion criteria of this study were as follows: (a) SCLC patients who have progressed after two or more lines of chemotherapy; (b) with BM at baseline; (c) without symptoms associated with BM; or (d) were stable after treatment for BM, not showing new or enlarged brain lesions 2 weeks after treatment (new or enlarged lesions appeared within 2 weeks after treatment indicating that they developed rapidly and were prone to present symptoms of BM), and stopped steroid or anticonvulsant therapy for at least 14 days before treatment initiation (the metabolism of anlotinib will be affected by anticonvulsant drugs such as phenytoin and carbamazepine).

### 2.2 Assessment

Computed tomography (CT) brain scans and enhanced magnetic resonance imaging (MRI) were performed on all patients during the screening period. The extracranial and intracranial lesions were evaluated based on Response Evaluation Criteria In Solid Tumors version 1.1 (RECIST 1.1) at baseline and during the treatment [20]. Adverse events (AEs) during the treatment were recorded and graded according to the National Cancer Institute Common Terminology Criteria for Adverse Events (NCI‐CTCAE) version 4.03.

### 2.3 Data collection and definitions

Baseline characteristics of patients were reviewed, including age, sex, Eastern Cooperative Oncology Group performance status, smoking history, previous treatment, and relapse pattern to chemotherapy.

Outcomes in this study included PFS, OS, time to central nervous system (CNS) progression, CNS objective response rate (ORR), CNS disease control rate (DCR), and safety profiles. The CNS response in target lesions (TLs) was classified as complete response (CR), partial response (PR), stable disease (SD), progressive disease (PD), or not evaluable (NE), while the CNS response in nontargeted lesions (NTLs) was categorized as CR, non‐CR/non‐PD (NCR/NPD), PD, or NE. Time to CNS progression was defined as the time from randomization to the first radiographic confirmation of CNS progression. The CNS ORR is determined by the numbers and percentage of patients with CNS response of CR or PR. CNS DCR was assessed by the numbers and percentages of patients with a therapeutic effect evaluation of CR, non‐CR, non‐PD, PR, or SD. PFS was defined as the time from randomization to disease progression or death from any cause, whichever came first. OS was defined as the time from randomization to death from any cause.

### 2.4 Statistical analyses

SAS v9.4 (SAS Institute) was utilized for all statistical analysis. Wilcoxon rank‐sum tests were used to compare the age of two groups, and Fisher's exact test was conducted for other categorical variables comparison. The Kaplan–Meier method was used for describing PFS and OS, and the log‐rank test was used to compare the two groups. To estimate the association between anlotinib and PFS, a multivariable Cox regression model (enter method) was used. Variables with statistical significance (*p* < 0.05) in the univariable analysis and clinical significance were included in the multivariable model. A competing risk methodology with a few modifications was used for assessing the cumulative incidence rates (CIR) of CNS progression and time to CNS progression. The possibility of CNS progression (with or without systemic progression), non‐CNS progression, or death was considered, and only the first event was included. The Pearson's *χ* ^2^ test or Fisher's exact test was utilized to compare CNS ORR and CNS DCR of two groups as appropriate. Two‐sided *p* < 0.05 indicated statistical significance.

## 3 RESULTS

### 3.1 Baseline characteristics of patients

From March 30, 2017, to June 8, 2018, 120 patients were enrolled in the ALTER1202 study, including 30 patients with BM at baseline (21 patients in the anlotinib group, and 9 patients in the placebo group). There are 13 and 9 males in the anlotinib group and in the placebo group, respectively. The median ages were 51 (43–68) years old and 61 (50–69) years old in the anlotinib group and placebo group, respectively (*p* = 0.0732). In the anlotinib and placebo groups, 17 cases (80.9%) and 6 cases (66.7%) had previously undergone brain radiotherapy (*p* = 0.0816). There are more current smokers in the placebo group than in the anlotinib group (*p* = 0.0159). Detailed information is presented in the Table 1.

**Table 1:** Baseline characteristics of patients

Variables	Anlotinib (*n* = 21)	Placebo (*n* = 9)	*p* Value
Age, year, median (range)	51 (42–67)	61 (49–68)	0.0732
Sex, male, *n* (%)	13 (61.9)	9 (100.0)	0.0665
ECOG performance status, *n* (%)			0.5172
0	1 (4.8)	0 (0.0)	
1	20 (95.2)	8 (88.9)	
2	0 (0.0)	1 (11.1)	
Smoking history, *n* (%)			0.0159
Never	10 (47.6)	0 (0.0)	
Current	10 (47.6)	9 (100.0)	
Former	1 (4.8)	0 (0.0)	
Previous lines of chemotherapy, *n* (%)			0.2096
2	13 (61.9)	8 (88.9)	
≥3	8 (38.1)	1 (11.1)	
Previous surgery, *n* (%)	2 (9.5)	0 (0.0)	1.0000
Previous extracranial radiotherapy, *n* (%)	17 (81.0)	8 (88.9)	1.0000
Previous brain radiotherapy, *n* (%)	17 (81.0)	4 (44.4)	0.0816
Other antitumor therapy, *n* (%)	6 (28.6)	2 (22.2)	1.0000
Relapse pattern to chemotherapy, *n* (%)			0.5345
Sensitive (relapse >3 month)	3 (14.3)	0 (0.0)	
Refractory (relapse ≤3 month)	18 (85.7)	9 (100.0)	

Abbreviation: ECOG, Eastern Cooperative Oncology Group.

### 3.2 Survival data

**Figure 1:**
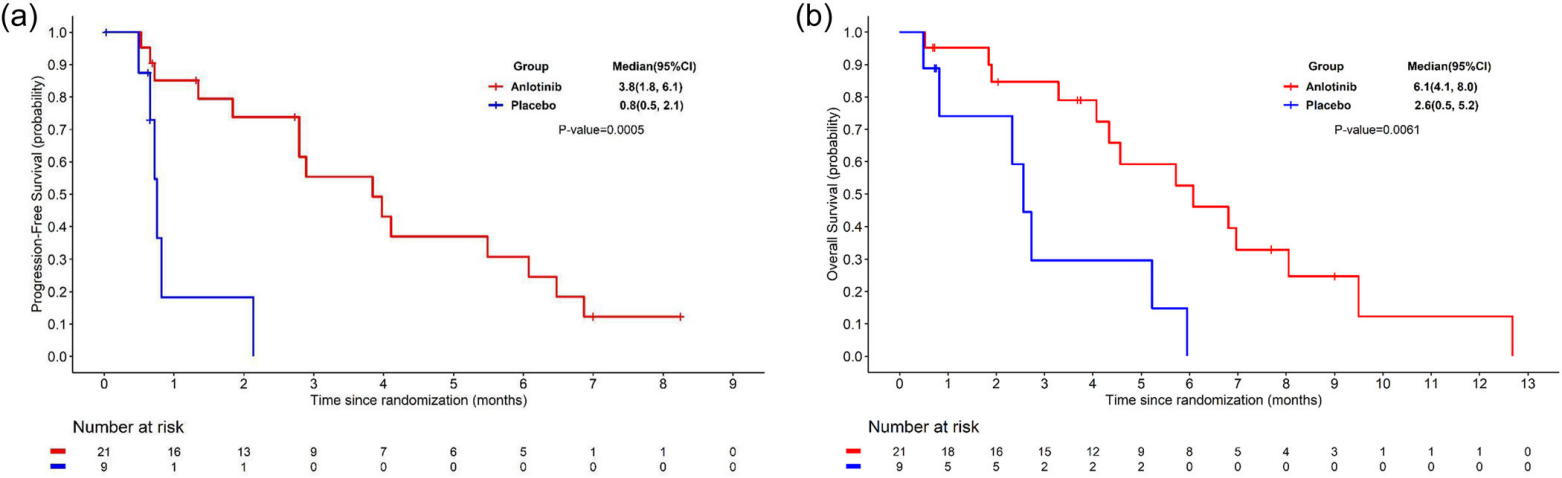
Kaplan–Meier curves of (a) progression‐free survival (b) overall survival in two groups

As of June 30, 2018, the median follow‐up was 9.0 (range: 0.49–12.68) months. A total of 15 and 6 patients administered anlotinib or placebo‐developed disease progression or died, respectively. The median PFS was 3.8 months (95% confidence interval [CI]: 1.8–6.1) in the anlotinib group, and 0.8 months (95% CI: 0.5–2.1) in the placebo group, respectively. The anlotinib group had a median OS of 6.1 months (95% CI: 4.1–8.0), and the placebo group had a median OS of 2.6 months (95% CI: 0.5–5.2). Anlotinib significantly prolonged median PFS (hazard ratio [HR] = 0.15, 95% CI: 0.04–0.51, *p* = 0.0005) and median OS (HR = 0.26, 95% CI: 0.09–0.73, *p* = 0.0061) in SCLC patients with BM (Figure 1).

Anlotinib was independently associated with PFS (HR = 0.17, 95% CI: 0.04–0.71, *p* = 0.0148) after adjusting for smoking history and previous brain radiotherapy (Table 2).

**Table 2:** Multivariable Cox regression analysis for the progression‐free survival

Variable	Adjusted HR (95% CI)	*p* Value
Anlotinib	0.17 (0.04–0.71)	0.0148
Smoking history		
Never	Reference	
Current	1.8 (0.59–5.44)	0.2992
Former	0 (0.00–NE)	0.9946
Previous brain radiotherapy	1.17 (0.36–3.81)	0.7908

Abbreviations: CI, confidence interval; HR, hazard ratio; NE, not evaluable.

### 3.3 Time to CNS progression and CNS response

**Figure 2:**
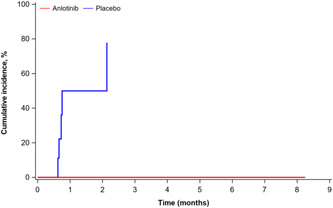
Cumulative incidence rate of central nervous system (CNS) progression. The median times to CNS progression were not reached in the anlotinib group and 0.8 months (95% confidence interval: 0.7–2.1) in the placebo group (*p* < 0.0001), respectively.

The median time to CNS progression was not reached in the anlotinib group, and 0.8 months (95% CI: 0.7–2.1) in the placebo group, respectively (Figure 2). In patients treated with anlotinib and placebo, the 2‐month CIR for CNS progression was 0 (95% CI: 0.0–0.0) and 50% (95% CI: 11.0–80.5), respectively. In comparison with placebo, anlotinib significantly prolonged the time to CNS progression (*p* < 0.0001).

Among all patients, the CNS ORR was numerically higher in the anlotinib group than the placebo group (9.5% vs. 0.0%), while the CNS DCR was significantly higher (95.2% vs. 22.2%, *p* = 0.0001). In patients with TLs, the CNS ORR was higher in the anlotinib group than in the placebo group (100% vs. 16.7%, *p* = 0.0152). Among patients with NTLs, the CNS DCR was 93.8% in the anlotinib group and 33.3% in the placebo group (*p* = 0.0506) (Table 3).

**Table 3:** Summary of CNS response

Response	Patients with TL		Patients with only NTL		Patients with BM (TL and NTL)	
	Anlotinib (*n* = 5)	Placebo (*n* = 6)	Anlotinib (*n* = 16)	Placebo (*n* = 3)	Anlotinib (*n* = 21)	Placebo (*n* = 9)
CR, *n* (%)	0 (0.0)	0 (0.0)	2 (12.5)	0 (0.0)	2 (9.5)	0 (0.0)
PR, *n* (%)	0 (0.0)	0 (0.0)	NA	NA	0 (0.0)	0 (0.0)
SD, *n* (%)	5 (100.0)	1 (16.7)	NA	NA	5 (23.8)	1 (11.1)
NCR/NPD, *n* (%)	NA	NA	13 (81.3)	1 (33.3)	13 (61.9)	1 (11.1)
PD, *n* (%)	0 (0.0)	4 (66.7)	0 (0.0)	1 (33.3)	0 (0.0)	5 (55.6)
NE, *n* (%)	0 (0.0)	1 (16.7)	1 (6.3)	1 (33.3)	1 (4.8)	2 (22.2)
ORR, *n* (%)	0 (0.0)	0 (0.0)	2 (12.5)	0 (0.0)	2 (9.5)	0 (0.0)
95% CI	–	–	(1.6–38.4)	–	(1.2–30.4)	–
*p*	–		1.0000		1.0000	
DCR, *n* (%)	5 (100.0)	1 (16.7)	15 (93.8)	1 (33.3)	20 (95.2)	2 (22.2)
95% CI	(47.8–100.0)	(0.4–64.1)	(69.8–99.8)	(0.8–90.6)	(76.2–99.9)	(2.8–60.0)
*p*	0.0152		0.0506		0.0001	

Abbreviations: CNS, central nervous system; CR, complete response; DCR, disease control rate; L, target lesion; NA, not applicable; NCR, non‐complete response; NE, not evaluable; NPD, non‐progressive disease, NTL, non‐target lesion; ORR, objective response rate; PD, progressive disease; PR, partial response; SD, stable disease.

### 3.4 Safety profiles

Eight patients (88.9%) in the placebo group and all patients (100%) treated with anlotinib experienced AEs. SAE occurred in eight patients (38.1% [95% CI: 18.1–61.6]) in the anlotinib group and two (22.2%, [95% CI: 2.8–60.0]) in the placebo group. In patients treated with anlotinib, the most common AEs were loss of appetite (47.6%), weight loss (42.9%), hypertriglyceridemia (38.1%), and leukopenia (38.1%), while the most common grade 3 or higher AEs were increased lipase (19.0%), hypertension (14.3%), and hyponatremia (14.3%) (Table 4). There were two AEs leading to death in the anlotinib group, which were deemed to be possibly unrelated to the treatment by the investigator. Both cases died after withdrawing from the study, and the causes of death were not recorded. One patient in the placebo group died due to AE.

**Table 4:** Adverse events

Events, *n* (%)	Anlotinib (*n* = 21)		Placebo (*n* = 9)	
	Any grade	Grade ≥3	Any grade	Grade ≥3
AE	21 (100.0)	13 (61.9)	8 (88.9)	2 (22.2)
SAE	8 (38.1)	–	2 (22.2)	–
AE leading to dose reduction	0 (0.0)	0 (0.0)	0 (0.0)	0 (0.0)
AE leading to treatment discontinuation	4 (19.0)	3 (14.3)	0 (0.0)	0 (0.0)
AE leading to death	2 (9.5)	–	1 (11.1)	–
AEs of any grade occurring ≥10% of patients or grade 3 or higher events occurring ≥2% in either group				
Loss of appetite	10 (47.6)	1 (4.8)	3 (33.3)	0 (0.0)
Weight loss	9 (42.9)	0 (0.0)	0 (0.0)	0 (0.0)
Hypertriglyceridemia	8 (38.1)	2 (9.5)	0 (0.0)	0 (0.0)
Leukopenia	8 (38.1)	0 (0.0)	0 (0.0)	0 (0.0)
Hypertension	7 (33.3)	3 (14.3)	0 (0.0)	0 (0.0)
Diarrhea	7 (33.3)	0 (0.0)	0 (0.0)	0 (0.0)
Sinus tachycardia	7 (33.3)	0 (0.0)	1 (11.1)	0 (0.0)
Hyponatremia	7 (33.3)	3 (14.3)	1 (11.1)	0 (0.0)
Elevated γ‐glutamyl transferase	7 (33.3)	2 (9.5)	1 (11.1)	1 (11.1)
Increased blood thyroid‐stimulating hormone	6 (28.6)	0 (0.0)	0 (0.0)	0 (0.0)
Decreased lymphocyte count	6 (28.6)	2 (9.5)	1 (11.1)	1 (11.1)
Hypercholesterolemia	6 (28.6)	0 (0.0)	0 (0.0)	0 (0.0)
Hypoalbuminemia	6 (28.6)	0 (0.0)	0 (0.0)	0 (0.0)
Increased lipase	5 (23.8)	4 (19.0)	0 (0.0)	0 (0.0)
Palmoplantar redness syndrome	5 (23.8)	0 (0.0)	0 (0.0)	0 (0.0)
Elevated aspartate aminotransferase	5 (23.8)	0 (0.0)	2 (22.2)	0 (0.0)
Fatigue	5 (23.8)	1 (4.8)	2 (22.2)	0 (0.0)
Urine red and white blood cell positive	5 (23.8)	0 (0.0)	0 (0.0)	0 (0.0)
Decreased platelet count	4 (19.0)	1 (4.8)	1 (11.1)	0 (0.0)
Increased blood alkaline phosphatase	4 (19.0)	0 (0.0)	1 (11.1)	0 (0.0)
Chest pain	4 (19.0)	0 (0.0)	0 (0.0)	0 (0.0)
Anemia	4 (19.0)	0 (0.0)	1 (11.1)	0 (0.0)
Vomiting	4 (19.0)	0 (0.0)	4 (44.4)	0 (0.0)
Expectoration	4 (19.0)	0 (0.0)	1 (11.1)	0 (0.0)
Nausea	4 (19.0)	0 (0.0)	1 (11.1)	0 (0.0)
Elevated alanine aminotransferase	4 (19.0)	0 (0.0)	2 (22.2)	0 (0.0)
Backache	4 (19.0)	0 (0.0)	1 (11.1)	0 (0.0)
Limb pain	3 (14.3)	0 (0.0)	0 (0.0)	0 (0.0)
Elevated blood bilirubin	3 (14.3)	0 (0.0)	1 (11.1)	0 (0.0)
ECG QT interval extension	3 (14.3)	0 (0.0)	1 (11.1)	0 (0.0)
Death	2 (9.5)	2 (9.5)	1 (11.1)	1 (11.1)
Urine red blood cell positive	3 (14.3)	0 (0.0)	0 (0.0)	0 (0.0)
Hypothyroidism	3 (14.3)	0 (0.0)	0 (0.0)	0 (0.0)
Hyperglycemia	3 (14.3)	0 (0.0)	1 (11.1)	0 (0.0)
Stomachache	3 (14.3)	0 (0.0)	1 (11.1)	0 (0.0)
Elevated amylase	3 (14.3)	2 (9.5)	1 (11.1)	1 (11.1)
Hard to swallow	2 (9.5)	1 (4.8)	0 (0.0)	0 (0.0)
Elevated direct bilirubin	2 (9.5)	1 (4.8)	0 (0.0)	0 (0.0)
Hypophosphatemia	2 (9.5)	1 (4.8)	2 (22.2)	0 (0.0)
Fremitus	0 (0.0)	0 (0.0)	1 (11.1)	0 (0.0)
Elevated creatine phosphokinase	0 (0.0)	0 (0.0)	1 (11.1)	0 (0.0)
Chest discomfort	0 (0.0)	0 (0.0)	1 (11.1)	0 (0.0)
Elevated fibrin d‐dimer	0 (0.0)	0 (0.0)	1 (11.1)	0 (0.0)
Influenza‐like illness	0 (0.0)	0 (0.0)	1 (11.1)	0 (0.0)
Oral ulcer	0 (0.0)	0 (0.0)	1 (11.1)	0 (0.0)
Hyperuricemia	0 (0.0)	0 (0.0)	1 (11.1)	0 (0.0)
Infectious pneumonia	0 (0.0)	0 (0.0)	1 (11.1)	0 (0.0)
Dysphonia	0 (0.0)	0 (0.0)	1 (11.1)	0 (0.0)
Hypokalemia	0 (0.0)	0 (0.0)	1 (11.1)	0 (0.0)
Constipation	0 (0.0)	0 (0.0)	1 (11.1)	0 (0.0)

Abbreviations: AE, adverse event; ECG, electrocardiograph; SAE, serious adverse event.

## 4 DISCUSSION

The development of BM results in poor prognosis in patients with SCLC. According to recent studies, immunotherapy prolongs OS in patients with SCLC, but survival in patients with SCLC with BM is still limited. This post hoc analysis of a multicenter, randomized, double‐blind, placebo‐controlled phase 2 study explored the efficacy and safety of anlotinib as a third‐line or above treatment for SCLC patients with BM. The results showed that anlotinib significantly prolonged PFS, OS, and time to CNS progression more than the placebo. As a consequence, anlotinib has been shown to demonstrate CNS activity in patients with SCLC and BM, as well as acceptable safety, and delays CNS progression significantly.

According to the present subgroup analysis, anlotinib improved PFS (median PFS: 3.8 vs. 0.8 months; HR = 0.15, 95% CI: 0.04–0.51; *p* = 0.0005) and OS (median OS: 6.1 vs. 2.6 months; HR = 0.26, 95% CI: 0.09–0.73; *p* = 0.0061) for SCLC patients with BM, with similar results in the overall population in the ALTER1202 study (median PFS: 4.1 vs. 0.7 months, HR = 0.19, 95% CI: 0.12–0.32, *p* < 0.0001; median OS: 7.3 vs. 4.9 months, HR = 0.53, 95% CI: 0.34–0.81, *p* = 0.0029) [19]. Further analysis revealed that anlotinib prolonged the time to CNS progression (*p* < 0.0001) and enhanced the CNS response (CNS DCR: 95.2% vs. 22.2%, *p* = 0.0001), which may contribute to the extension of the PFS and OS after anlotinib administration. Based on a previous subgroup analysis, anlotinib prolongs PFS (HR = 0.29, 95% CI: 0.15–0.56), and increases time to brain progression (TTBP) (HR = 0.11, 95% CI: 0.03–0.41, *p* = 0.001) in NSCLC patients with BM, which was consistent with our findings [17]. It has also been reported that anlotinib was effective against BM from lung cancer in two cases [21]. In summary, these findings suggest anlotinib may inhibit intracranial tumor growth in BM. The activities of cancer cells are determined by their proximity to a blood vessel [22]. By inhibiting angiogenesis, anlotinib exhibits promising antitumor effects in advanced NSCLC, medullary thyroid cancer, and metastatic renal cell cancer [17, 23, 24]. Anlotinib suppresses tumor cell proliferation by inhibiting VEGFR 1–3, FGFR 1–4, and PDGFR α and β [16]. During the tumor progression, a disruption in the blood–brain barrier (BBB) can lead to the formation of the blood–tumor barrier (BTB), which is more permeable than the BBB [25]. Besides, hydrophobic molecules with molecular weights of less than 500 Da were able to cross the BBB/BTB [26]. Anlotinib has a molecular weight of 480.36 Da, and it is a hydrophobic small molecule under neutral conditions. Anlotinib may therefore be able to cross the BTB and inhibit disease progression. Further research is needed to understand how multitargeted antiangiogenic agents exert anti‐BM effects.

Several treatment options, including WBRT, SRT, and immunotherapy, alone or in combination, are available for patients with BM [27], while the prognosis in cancer patients with BM is still dismal. With advances in molecular profiling, it is possible to determine which treatments are the most effective for BM [27]. In the IMpower‐133 trial, atezolizumab did not confer any benefit to BM (HR = 1.07, 95% CI: 0.47–2.43) [15]. Furthermore, the CASPIAN study failed to demonstrate that durvalumab combined with chemotherapy significantly improved the survival of patients with BM (HR = 0.69, 95% CI: 0.35–1.31) [14]. Whether immunochemotherapy is beneficial to BM patients requires more research. According to a review of 16 studies, TKIs can be effective in controlling BM from NSCLC, especially in those with mutations in EGFR [28]. Anlotinib combined with radiotherapy is currently being studied and might provide valuable treatment options for patients with BM [29].

This study had some limitations. First, this was a post hoc analysis of a randomized controlled trial. A completely balanced baseline characteristic was not achieved as a result of the randomization effect not being maintained. In this post hoc study, most baseline characteristics did not differ significantly between the two groups, while the significant difference in smoking history (*p* = 0.0159) was adjusted in the multivariable model. Furthermore, the statistical power of this study was likely insufficient due to the limited sample size.

## 5 CONCLUSIONS

In this post hoc analysis of a multicenter, randomized, double‐blind, placebo‐controlled trial, anlotinib demonstrated higher CNS response and potential survival benefit in SCLC with BM compared with placebo, with manageable toxicity. The evidence of TKIs in SCLC patients with BM was limited now, and our results provided a potential treatment option for heavily pretreated patients with BM. Further randomized controlled trials with larger sample sizes are needed to confirm our conclusion.

## AUTHOR CONTRIBUTIONS

**Ying Cheng**: Conceptualization; methodology; data curation; formal analysis; supervision; original draft; review; editing of the manuscript. **Qiming Wang**: Data curation; formal analysis; manuscript review. **Kai Li**: Data curation; formal analysis; manuscript review. **Jianhua Shi**: Data curation; formal analysis; manuscript review. **Baohui Han**: Data curation; formal analysis; manuscript review. **Lin Wu**: Data curation; formal analysis; manuscript review. **Gongyan Chen**: Data curation; formal analysis; manuscript review. **Jianxing He**: Data curation; formal analysis; manuscript review. **Jie Wang**: Data curation; formal analysis; manuscript review. **Haifeng Qin**: Data curation; formal analysis; manuscript review. **Xiaoling Li:** Data curation; formal analysis; manuscript review.

## CONFLICT OF INTEREST

The authors declare no conflict of interest.

## ETHICS STATEMENT

The study followed the Declaration of Helsinki as well as the Good Clinical Practice Guidelines. This trial had approval from the institutional review board of each participating institution (approval number: 201701‐002‐01).

## INFORMED CONSENT

All patients provided signed informed consent.

## Data Availability

The data sets utilized and/or assessed in this study are available from the corresponding author upon reasonable request.
